# Supplementary material for: Quantum bath suppression in a superconducting circuit by immersion cooling
Source: Nat Commun. 2023 Jun 14;14:3522. doi: 10.1038/s41467-023-39249-z (PMC10267208; doi:10.1038/s41467-023-39249-z)
Supplement: Supplementary file 1 — Supplementary Information [file 41467_2023_39249_MOESM1_ESM.pdf]

Supplementary Information: Quantum bath suppression in a superconducting circuit by immersion cooling

M. Lucas[1], A. V. Danilov[2], L. V. Levitin[1], A. Jayaraman[2], A. J. Casey[1], L. Faoro[3], A. Ya. Tzalenchuk[1,4], S. E. Kubatkin[2], J. Saunders[1], and S. E. de Graaf[4]

[1] *Physics Department, Royal Holloway University of London, Egham, United Kingdom*
[2] *Department of Microtechnology and Nanoscience MC2, Chalmers University of Technology, SE-412 96 Göteborg, Sweden*
[3] *Google Quantum AI, Google Research, Mountain View, CA, USA and*
[4] *National Physical Laboratory, Teddington TW11 0LW, United Kingdom*

Experiments on quantum circuits at microkelvin temperatures is a cross-disciplinary effort. In this Supplementary we provide a detailed account of the cryogenic, microwave, metrology, and analytical background of our research in order to catalyze future development in this direction.

CONTENTS

1. Experimental setup 1
   A. Refrigerator and thermometry 1
   B. Microwave wiring 1
   C. The experimental cell 3
   D. Temperature of the helium bath 4
   E. The solenoid for electron spin resonance measurements 5

2. Measurements 5

3. Noise analysis 5

4. Extended data 6
   A. Full noise data 6
   B. Power dependence of quality factor 6
   C. Dielectric loss due to ^{3}He 7
   D. Cooling in the presence of a thin ^{3}He film 10
   E. Pressure dependence 10
   F. Additional data on noise at sub-mK temperatures 11

5. Theoretical models 11
   A. Temperature dependence of the noise 11
   B. Power dependence of the quality factor 14

References 14

1. EXPERIMENTAL SETUP

A. Refrigerator and thermometry

The experiment was conducted on a cryogen-free Triton 200 Oxford Instruments dilution refrigerator (DR). This DR has been fitted with an adiabatic nuclear demagnetisation stage that allows it to operate at temperatures as low as 400 µK. This system will be referred to as ND4 and is described in detail in [1].

The experiment is installed in the ultra-low RF-noise environment of ND4, on the adiabatic nuclear demagnetisation refrigerator plate (ANDRP), allowing it to be cooled below 1 mK.

In addition to the RuO_2 thermometer on the mixing chamber plate (MCP), ND4 is also equipped with two current sensing noise thermometers (CSNT) with SQUID readout: one on the MCP and one on the ANDRP. CSNT allows to accurately measure temperatures down to 100 µK [2, 3]. We used a low resistance sensor ($\approx 2\,\mathrm{m\Omega}$) for the ANDRP CSNT to reduce the error on the measured temperature originating from the background noise of the SQUID. The temperature is obtained by fitting the power spectral density averaged over fifty noise traces. Each noise trace is sampled 2^{20} times at 200×10^3 samples/s, which gives an acquisition time of 5.24 s per trace and a temperature measurement every 262 s. This brings the error in temperature measurement below 1 % at 0.4 mK and below 0.1 % at 10 mK.

B. Microwave wiring

The microwave installation in the cryostat used for the measurements was built following the recipe detailed in [4], extended for operation compatible with temperatures below 1 mK. Supplementary Fig. **1** shows an overview of the whole microwave assembly, which consists of one input line and one output line. UT-034 (0.86 mm OD) NbTi-NbTi semi-rigid superconducting cryogenic coax cables are used for the connections between the cell and the HEMT amplifier on the 4 K plate, and UT-034 CuNi-CuNi semi-rigid cryogenic coax cables elsewhere.

Both lines are thermalised to each temperature stage with attenuators (the choice of attenuation will be discussed later). Infra-red (IR) frequencies are filtered out from both lines with an infra-red eccosorb filter, which is thermalised to the MCP. The output signal is amplified with a cryogenic HEMT amplifier (LNF-LNC4_8C) with a gain at 4 K of 41 dB and a noise temperature at 6 GHz of 1.5 K, installed on the pulse tube stage 2 plate (PT2P) at 4 K. To prevent thermal radiation from the HEMT reaching the experiment without attenuating the outgoing signal, a triple junction isolator (LNF-ISISISC4_8A) with $S_{21}(6\,\mathrm{GHz}, 3\,\mathrm{K}) = -0.1\,\mathrm{dB}$ and an

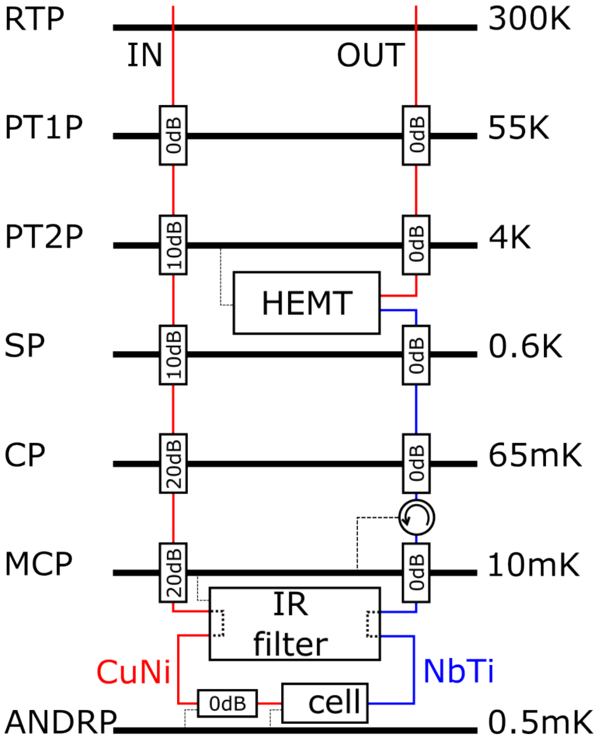

Supplementary Fig. 1. Microwave installation used for the present experiment. 'RTP': room temperature plate, 'PT1P': pulse tube stage 1 plate, 'PT2P': pulse tube stage 2 plate, 'CP': cold plate (or 100 mK plate), 'MCP': mixing chamber plate, 'ANDRP': adiabatic nuclear demagnetisation refrigerator plate. Red(blue) lines represent the CuNi-CuNi(NbTi-NbTi) UT-034 coax lines. The smaller rectangular boxes show the configuration of attenuators. 'IR filter' is a eccosorb infrared filter. The circle with the circular arrow between MCP and CP is a triple-junction isolator. 'HEMT' is a high electron mobility transistor amplifier. The dotted lines represent the thermalisation link to the different temperature stages.

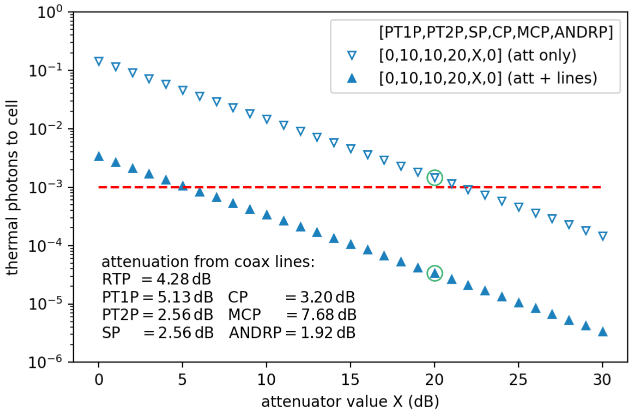

Supplementary Fig. 2. The population of thermal photons at 6 GHz reaching the cell for different values of the input line MCP attenuator. The empty markers show the population of thermal photons calculated without taking into account of the attenuation in the lines, as an upper bound. The solid markers show the population of thermal photons calculated taking also the attenuation in the coaxial cables into account. The temperatures of the coaxial lines are assumed to be their high-temperature end. The values (in dB) of the attenuators on the other plates are given in the label. Choosing a value of 20 dB provides a sufficiently low enough amount of thermal photons to the cell (green circles).

isolation at $(6\,\mathrm{GHz}, 3\,\mathrm{K}) \geq 70\,\mathrm{dB}$ is placed between the MCP and the still plate (SP).

The amount of attenuation needed is determined by how low one requires the population of thermal photons to be at the experiment and the distribution of the attenuation, as well as the choice of the microwave components that need to be carefully chosen for compatibility with the operation of an adiabatic nuclear demagnetisation refrigerator. For a typical qubit experiment a population of thermal photons at 6 GHz well below 10^{-3} is desired [4]. In order to achieve that, one needs at least 60 dB attenuation on the input line. The most effective way to reduce the amount of thermal photons is to install a significant amount of the attenuation on the coldest plate of the fridge, but that also leads to excessive dissipation of heat. Therefore, the attenuators on the drive line have been distributed between the different stages (see Supplementary Fig. **1**). To thermalise the central conductor in the coax lines, 0 dB attenuators were used where no attenuation was needed; this includes all stages for the output line, and pulse tube stage 1 plate (PT1P) and ANDRP for the input line. Here we further choose to have 0 dB attenuation on the input line at the ANDRP to minimise the active heat load. In Supplementary Fig. **2** we show that with 20 dB on the MCP stage we are still able to achieve a thermal photon population at 6 GHz below 10^{-3}.

The passive (due to thermal conductivity) and active (RF signals) thermal loads of the RF-setup on the stages of the DR are estimated with a simplified thermal model, which provides an upper-bound for the thermal load. In this model the attenuation in an input cable connecting two different stages of the DR, is added to the attenuator connected to its low-temperature end. The attenuation in the input cables connecting the MCP to the IR filter and the ANDRP attenuator to the cell are added to the attenuators on the MCP and ANDRP, respectively. This is because these two cables are at the same temperature as their respective stages. In the present model only the passive load is considered in the output line. This is justified by the low-power level of the output signal exiting the cell (below $\leq -85\,\mathrm{dBm}$ up to PT1P and $\leq -45\,\mathrm{dBm}$ after amplification at PT1P) and the negligible attenuation in the NbTi-NbTi cables. The combined loads on the CP and the MCP are less than 2 µW and 10 nW, respectively. Supplementary Fig. **3** also shows a total load to the ANDRP lower than 20 pW, which represents 0.5 % of the typical residual heat leak to the ANDRP.

To provide a best estimate for the photon number in the resonators, which main uncertainty stems from the

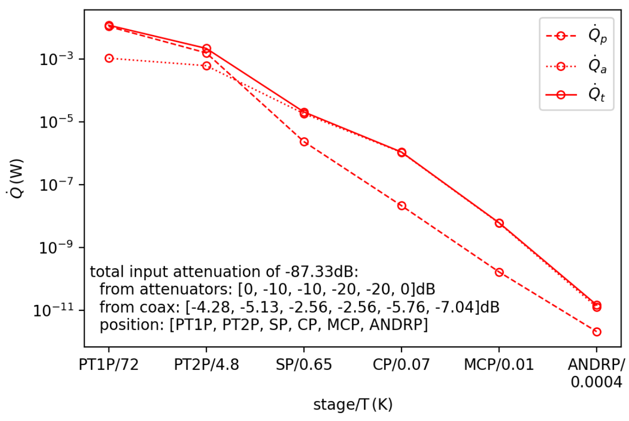

Supplementary Fig. 3. The passive and active loads coming from the RF-system to the different stages of the DR. The values used for the thermal conductivities were taken from [5] and references therein, and the fit functions where extrapolated to the lower temperatures, hence overestimating the values for the passive loads. The description of how the attenuation in the cables is divided across the attenuators on the different stages is given in the main text. The active load is computed for an input signal power of $P_{\text{in}} = 2.33\,\text{dBm}$, which provides $P_{\text{cell}} = -85\,\text{dBm}$ (the maximum operating power of the resonator) at the input of the cell.

determination of the input power to the sample chip at the low temperature stage, we measure the attenuation of the microwave input line at room temperature and correct for the change in coaxial cable attenuation due to cooling reported in the manufacturer's data sheet.

C. The experimental cell

Supplementary Fig. **4** shows an exploded view of the experimental cell, which is composed of three main elements: the sample holder, the sample cover and the lid. Each of these elements is itself composed of different parts, fulfilling specific functions.

Sample holder. The body of the sample holder is made of oxygen-free high conductivity (OFHC) copper. The body has a step-shaped profile at its base for the indium seal and is directly thermalised to the ANDRP and provides thermal anchoring to all the other components of the cell. The body is drilled through to receive three microwave feed-through connectors (shown in Supplementary Fig. **4**). These connectors are commercially available low-temperature hermetic feed-throughs. Two of the connectors are for the high-frequency in/out signals, and a third connector for a provisional DC-line. The sample is glued to the sample holder copper base with GE varnish and maintained with two BeCu clamps (as shown in Supplementary Fig. **4**). However, based on the thermal propertied of the materials involved (sapphire substrate and NbN conductor), negligible cooling is expected from thermal conduction through the mechanical anchors.

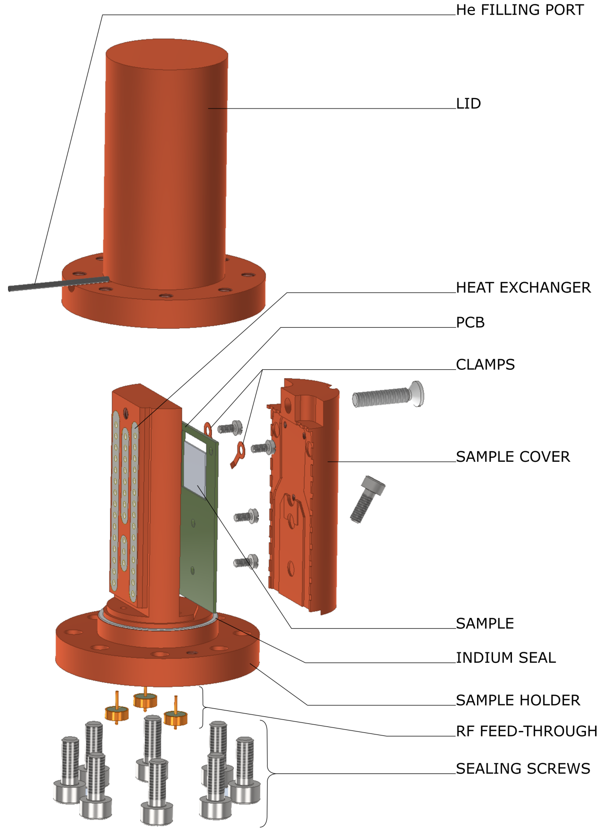

Supplementary Fig. 4. Exploded view of the He immersion cell used in the experiment. The lid, together with the base of the sample holder, the hermetic feed-through connectors and the indium seal, forms the leak tight enclosure of the immersion cell and is attached to the sample holder base with the eight sealing screws. A copper thermal link screwed in the base of the sample holder attaches and thermally connects the immersion cell to the ANDRP.

Heat exchanger. Because of the Kapitza boundary resistance, it is necessary to include heat exchanger in the immersion cell in order to cool down ^{3}He below 1 mK. Although the microscopic mechanisms influencing the the Kapitza resistance is not fully understood, there are well established recipes for how to improve thermal conductance of copper/liquid helium interfaces using silver sinter heat exchangers. Such a silver sinter heat exchanger has been made directly on the back of the sample holder, as shown in Supplementary Fig. **5**. Holes have been drilled in the sinter to satisfy a rule of thumb, which states that that there should no be more than 1 mm of sinter between bulk liquid helium and bulk copper. A surface area of 24.1 m^2 of the sinter has been measured with the BET (Brunauer–Emmett–Teller) N_2 isotherms method performed at 77 K. We use silver powder with a 70 nm grain size, giving an estimated Kapitza boundary resistance for this heat exchanger at the ^{3}He/heat exchanger interface of [5]

$$R_{\text{K}}(T) = 41.5\,T^{-1}\,\text{K/W}. \tag{1}$$

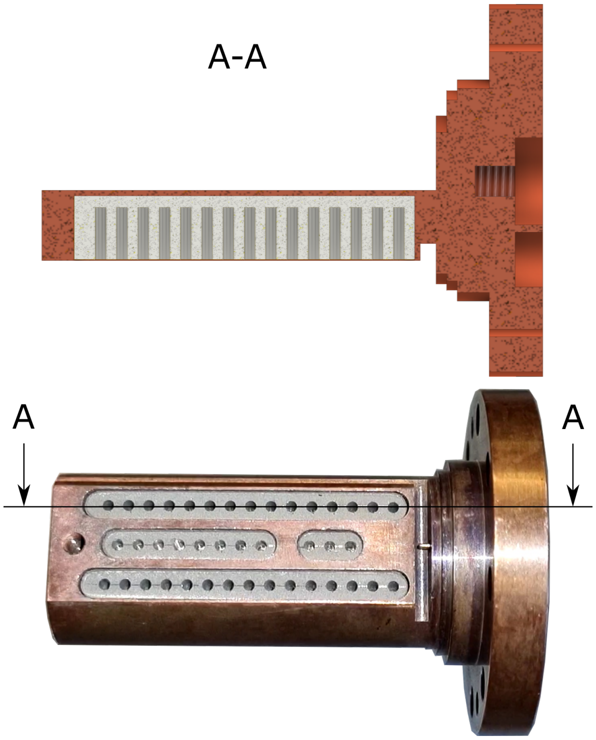

Supplementary Fig. 5. Silver sinter heat exchanger used for the thermalisation of the liquid ^{3}He, shown in grey on the CAD view at the top and on the photo at the bottom. The photo shows the back side of the sample holder with the four sinter pockets composing the heat exchanger of the immersion cell. Further details in the main text.

During the experiment, the immersion cell was filled with $n_{^3\mathrm{He}} = 0.1\,\mathrm{mol}$ of liquid ^{3}He, which has a heat capacity of

$$C(T)_{^3\mathrm{He}} = 2.3\,T\,\mathrm{J/K}, \tag{2}$$

up to 10 mK [5]. This gives a time constant for cooling the ^{3}He in the cell of

$$\tau = R_\mathrm{K} C_{^3\mathrm{He}} = 95\,\mathrm{s}. \tag{3}$$

Sample cover. The sample cover is made of OFHC copper and has two main functions: it serves as a displacement volume to reduce the amount of bulk liquid of ^{3}He inside the cell and it forms a RF-cavity around the sample. Minimising the amount of ^{3}He required to fill the cell means that the dimensions of the cavity around the sample must be carefully designed to prevent interfering box resonance modes.

Lid and ^{3}He fill line. The lid, made of OFHC copper, is attached to the base of the sample holder with eight stainless steel screws and an indium seal makes the cell leak tight. The total helium volume inside the cell is $3.98\,\mathrm{cm}^3$ and the volume is connected to the room-temperature ^{3}He gas handling system via a 140 µm inner-diameter (0.5 mm outer-diameter) CuNi capillary. To condense and thermalise the ^{3}He injected in the cell, at each stage of the DR the fill line is winded and silver-brazed around a $\frac{1}{4}$" copper tube, which is clamped to the cryostat plate.

To prevent pressure changes in the cell that would result from variations in the temperature profile along the fill line, a 1 l ballast volume is connected to the fill line at room temperature. This ballast volume reduces the impact of the density changes of ^{3}He, which below pressures of 5 bar has a strong temperature dependence above 2 K [6]. Hence the density of the ^{3}He is particularly sensitive to the temperature of the PT2P, which can fluctuate by up to 20 % during normal operation for various reasons (ramping of the demagnetisation magnet, fluctuations of the cooling water temperature in the compressor, etc.). The gas contained in the ballast volume can be considered as ideal in constant volume and its pressure thus scales with its temperature. At the time of the experiment the typical variation of the room temperature over 24 hours was about 2 °C around an average of 25 ° C, which gives a typical pressure stability of ≈ 0.7 %.

D. Temperature of the helium bath

There is no thermometer in our set up which directly measures the liquid ^{3}He temperature in the cell. Instead, we measure the temperature of the ANDRP with a CSNT. Because of non-zero thermal resistance, a temperature gradient appears along the thermal path between the ^{3}He bath and the ANDRP. In order to give an upper bound on the temperature difference between the plate and the ^{3}He in the steady state, we make the overestimated assumption that the sample (in operation) dissipates $\dot{Q} = 50\,\mathrm{pW}$ directly into the ^{3}He bath (this is 10 times more than what is expected as a result of any measurement) and that the sintered heat exchanger has the same temperature as the copper sample holder.

The temperature of the heat exchanger T_hx can be obtained from the Wiedemann-Franz law:

$$\dot{Q} = \frac{L_0}{2R}(T_\mathrm{hx}^2 - T_\mathrm{adnr}^2), \tag{4}$$

where $L_0 = 2.44 \times 10^{-8}\,\mathrm{W}\,\Omega\,\mathrm{K}^{-1}$ is the universal Lorentz number, $R = 1\,\mu\Omega$ is an upper bound of the electrical resistance along the thermal path from the heat exchanger to the ANDRP, and T_adnr is the temperature of the ANDRP. For $T_\mathrm{andr} = 400\,\mu\mathrm{K}$, this gives

$$T_\mathrm{hx} = \sqrt{T_\mathrm{andr}^2 + \frac{2R\dot{Q}}{L_0}} = 405\,\mu\mathrm{K}. \tag{5}$$

The temperature step at the ^{3}He/heat exchanger interface is given by $\dot{Q}R_\mathrm{K}(T) = \Delta T$, where $R_\mathrm{K}(T)$ is the temperature-dependent Kapitza boundary resistance given above and $\Delta T = T_{^3\mathrm{He}} - T_\mathrm{hx}$ is the positive temperature difference between the ^{3}He bath and the heat exchanger. Considering $R_\mathrm{K}(T_\mathrm{hx} = 405\,\mu\mathrm{K}) = 102500\,\mathrm{KW}^{-1}$, we arrive at $T_{^3\mathrm{He}} = 410\,\mu\mathrm{K}$; the temperature difference between the ANDRP and the liq-

uid ^{3}He bath is less than 2.5 % at the coldest temperature ($T_{\mathrm{andr}} = 400\,\mu\mathrm{K}$) and drops to less than 0.1 % at $T_{\mathrm{andr}} = 1\,\mathrm{mK}$.

As another example we take the case of $N \sim 300$ used in the noise measurements in the main manuscript which shows clear temperature dependence down to 1 mK. The circulating power in the resonator is $P_{\mathrm{circ}}(N) = N\hbar\omega_0^2$, and the amount of power dissipated into the TLS bath is given by

$$\dot{Q}_{\mathrm{diss}}(N) = N\frac{\pi\hbar\omega_0^2}{2Q_i(N)}. \tag{6}$$

This gives a dissipation $\dot{Q}_{\mathrm{diss}} \approx 3$ fW into the TLS bath, which is taken away by the ^{3}He. 3 fW is about 10^6 times smaller than the total heat leak to the nuclear stage of the fridge, and 1000 times smaller than the maximum experimental power assumed in the above estimate of the temperature gradient.

E. The solenoid for electron spin resonance measurements

The solenoid for the electron spin resonance (ESR) experiment was designed to be compatible with the design of the immersion cell and with a targeted field-to-current ratio around 200 mT/A. The precise value of the latter has been deduced from the ESR signature of the free-electron spins and is 220(2) mT/A. The solenoid was wound with a 107 µm single filament NbTi superconducting wire in CuNi cladding around a copper base with inner diameter of 24 mm and a wall thickness of 0.6 mm. For practical reasons, the ESR solenoid was not operated in persisted mode, as the ESR field was adjusted every tens of seconds. In order to prevent noise from room temperature instruments and power supplies to affect the magnetic field, a very low-pass filter with a cut-off frequency at -3 dB of $f_{\mathrm{c}} \approx 20$ mHz was used.

2. MEASUREMENTS

Here we provide additional information regarding the measurement setup and procedures. Two samples were measured, the first one containing two resonators (Resonator A and B) of design as described in [7]. The second chip with Resonators C, D, E (of design [8]) was measured in a consecutive cooldown in the same immersion cell. Samples were fabricated in the same way, using NbN on sapphire. Most notable difference between designs was that the second chip (C, D, E) had a in-plane interdigitated capacitor gap and superconductor strip width of 2 μm, compared to 1 μm for Resonators A and B. This is to somewhat reduce the coupling strength to individual TLS while still predominantly probing surface TLS. Though resonators A and B were frequency tunable, this functionality was not used in the present experiments. Both the magnitude of the noise and the single photon loss in the two resonator designs were roughly the same.

We measure the frequency fluctuations of the resonators using the well-established Pound-locking technique [9]. This technique is based on a single phase-modulated RF signal being passed down the measurement line in the cryostat. Importantly, the modulation frequency is chosen (here 1.3 MHz) such that phase modulation sidebands are outside the resonator bandwidth. When this signal passes through the sample and after detection in a square-law detector (diode), it can be shown that the contributions from the lower and upper sidebands cancel due to symmetry if the carrier signal frequency is on resonance with the resonator ($f = \nu_0$). However, if not on resonance there will exist a low frequency component after the detector at the phase modulation frequency. The measurement electronics are configured to detect and null this error signal by adjusting the carrier frequency in a feedback loop. An advantage of this method for cryogenic measurements is that both signal and reference are passed through the same RF cables, and instabilities due to e.g. temperature drift at room temperature and inside the cryostat, vibrations or other sources that can affect the signal propagation are suppressed.

Supplementary Fig. **6** shows the schematics of the Pound-locking electronics used. As a stable frequency reference we use a SRS FS740 rubidium frequency standard, that also serves as the frequency counter to determine the instantaneous resonance frequency ν_0. Using a gate time of 0.05 s we record the gap-free time series of $\nu_0(t)$, that we analyse (see below) to extract the $1/f$ noise magnitude. A SMB100A RF generator is used to produce the carrier frequency that is modulated using an analogue phase modulator, a ~ 1 MHz phase modulation signal is supplied by a Zurich Instruments HF2LI lock-in amplifier. We tap off part of the signal from the carrier generator and downconvert it using a second generator (Hittite hmc-t2220) to about 70 MHz, within the bandwidth of the counter. After amplification, filtering and detection in a RF diode the lock-in amplifier is used to demodulate the signal at the phase modulation frequency, and a PID controller (SRS SIM960) is given the task of nulling the lock-in signal by changing the frequency of the carrier generator operated in external frequency modulation mode.

3. NOISE ANALYSIS

To measure the noise we record the resonator center frequency $\nu_0(t)$ with a gap-less sampling rate $\delta t = 0.05$ s. We transfer sampled blocks of 1000 data points from the frequency counter and for each such transfer we also record the ANDRP temperature, i.e. every 50 s. For long measurements versus temperature we then divide the recorded dataset into smaller chunks, each corresponding to some temperature interval $\langle T\rangle \pm \delta T$ where

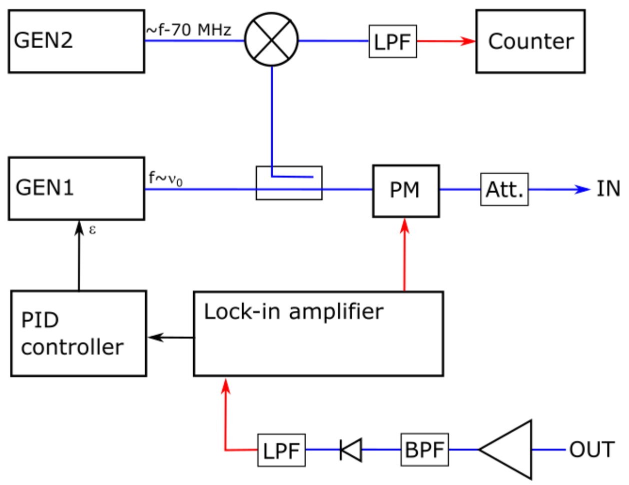

Supplementary Fig. 6. Simplified schematic of the Pound measurement setup used to measure frequency noise. LPF is a low pass filter, BPF is a 40 MHz wide frequency tunable band pass filter, PM is a phase modulator (HMC538LP4), and Att. is a tunable attenuator (Vaunix LDA-5018V). Blue lines indicate high frequency (GHz) signals, red lines low frequency (MHz) and black very low frequency (<kHz) signals.

the error bar in temperature is taken as the maximum and minimum deviation from the mean in the interval.

The sampled $\nu_0(t)$ signal is converted to frequency noise spectral density S_y by calculating the overlapping Allan-variance $\sigma_y^2(\tau)$ (AVAR) for M discrete samplings $\nu_k(n\tau)$ at multiples n of the sampling rate τ.

$$\sigma_y^2(n\tau) = \frac{1}{2(M-1)} \sum_{k=1}^{M-1} (\nu_{k+1} - \nu_k)^2. \tag{7}$$

For $1/f$ noise the power spectral density $S_y(f) = h_{-1}/f$ relates to the Allan variance as $\sigma_y^2 = 2\ln(2)h_{-1}$, where $h_{-1} = A_0/2\pi$ [10], and A_0 is the magnitude of the $1/f$ noise. The AVAR is evaluated at several time-scales $t = n\tau$ ranging from 10^0 to 10^3 seconds and h_{-1} is obtained by averaging the calculated AVAR across at least two decades in τ. Error bars in S_y are calculated from the standard deviation of the AVAR in the same time interval. The selected range varies somewhat from measurement to measurement, however it is not changed within a measurement. The reason for this is that different resonators and different conditions may lead to other noise mechanisms (such as white noise) entering at short timescales, or drift at very long timescales. Occasionally very strong individual TLS appear and temporarily affect the AVAR data (temporal drift). Because of this we adapt the range of times for analysis in order to avoid over-estimating the noise. Admittedly, this approach works less well at low measurement powers, where strong TLS appear more frequently and on many different timescales during measurements.

4. EXTENDED DATA

A. Full noise data

In Supplementary Fig. **7** we show the complete AVAR spectra behind the extracted data in Figure 3a in the main manuscript. We plot the AVAR for each time-bin that corresponds to a specific temperature range as the temperature was ramped up. Rather than ambiguously trying to fit and subtract these from the overall flat background, or picking different background levels circumventing some of the random telegraph noise (RTN), we have instead included these in the analysis and calculation of the average noise level presented in Figure 3 in the main manuscript. Here a large error bar is the result of a strong RTN fluctuator being present.

In Supplementary Fig. **9** we also show the measured temperature dependence of the noise from the second sample, resonator D, when the cell is filled with ^{3}He. The noise measurements take a significant amount of time ($\sim$ 6 days per temperature ramp carried out at one drive power) and therefore we have only measured noise in some of the resonators. The noise in resonator D is following the same general trend as for resonators A and B. Because of the larger capacitor gap in resonator D compared to resonators A and B the magnitude of the noise is somewhat lower.

Supplementary Table **2** summarises the fitted temperature dependence of the noise from multiple measurements conducted, both in the high temperature regime (above T_x) and low temperature regime (below T_x).

In Supplementary Fig. **8** we also present the same data as in Figure 3a of the main manuscript, without the 20x scaling applied to the in-vacuum data presented in Figure 3a in the main manuscript. Here we only retain a small scaling factor of $\sqrt{N^{\text{empty}}/N^{\text{full}}} = 1.29$ due to the small difference in photon number arising from the difference in Q_i with full and empty cell (measurements were conducted with the same applied power to the sample).

B. Power dependence of quality factor

Supplementary Fig. **10** and **11** show the internal resonator quality factor versus the average photon number for different resonators on the two samples measured. We note that a factor ~1000 times enhancement in saturation power as a result of ^{3}He filling was achieved on the second sample, and for the first sample the enhancement is somewhat smaller, 200-300 times. This can be due to a number of reasons, most likely due to the interface between ^{3}He and TLS medium being not exactly the same. As the samples were fabricated at different times we may expect different ageing and surface contamination. It could also be related to possible presence of a (sub-)monolayer of ^{4}He on the surface of resonators A and B. In the second cooldown (resonators, C, D and E) measures were taken to minimise ^{4}He contamination fur-

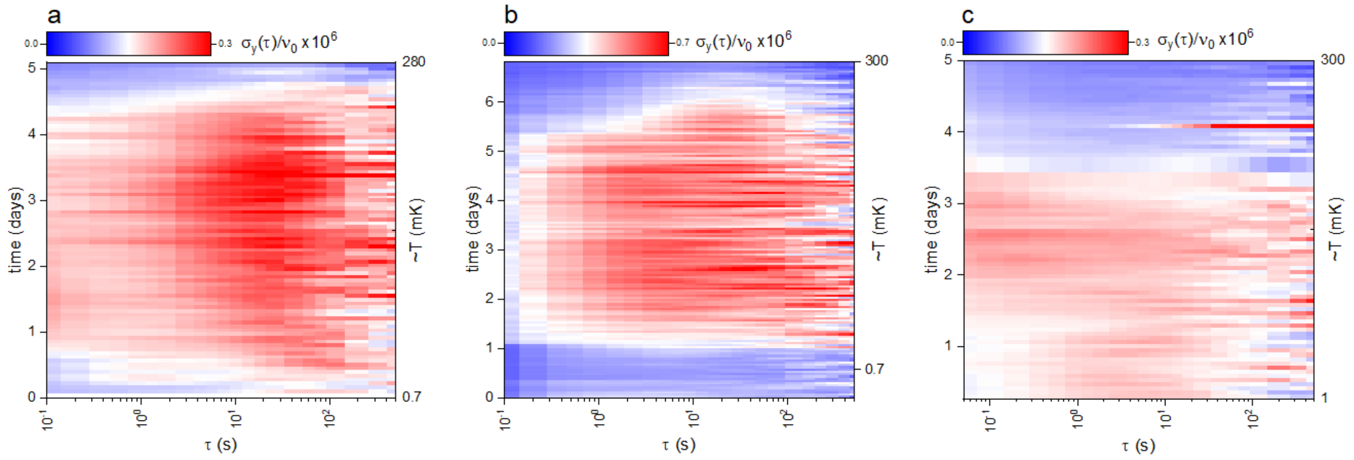

Supplementary Fig. 7. The calculated $\sigma_y(\tau)$ for the whole temperature ramp in Figure 3a in the main manuscript for a) Full cell $N \sim 400$, b) Full cell $N \sim 20$ and c) Empty cell $N \sim 400$, highlighting the temporal drift of individual TLS at different timescales. Color scale is magnitude of the AVAR $\sigma_y(\tau)$ with blue corresponding to low values and red to higher values (note the different color scales in panels a-c).

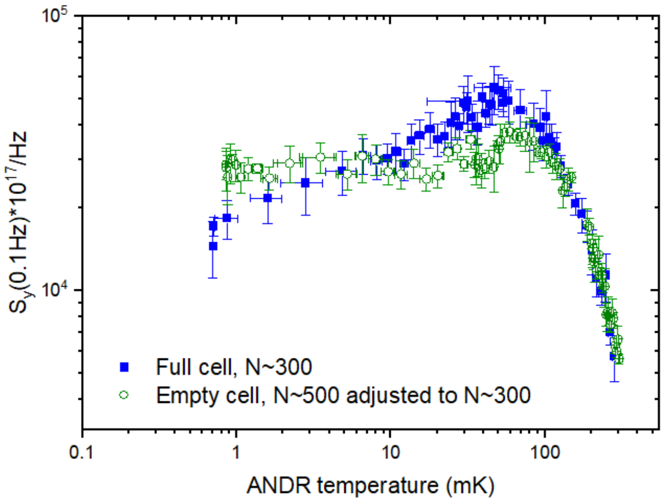

Supplementary Fig. 8. The same data as in Figure 3a in the main manuscript without the 20x scaling of the noise for the empty cell data. Both measurements were conducted with the same input power to the sample, which results slightly different photon numbers because of the change in Q. The corresponding scaling factor 1.29 has been applied here to compare the data at the same photon numbers. Error bars are defined as described in Supplementary Note 3.

ther. We note that the different capacitor gap of the two samples is expected to contribute in the opposite way.

C. Dielectric loss due to ^{3}He

The dielectric constant of ^{3}He is known to be $\varepsilon_r = 1.0426$ at GHz frequencies and saturated vapor pressure. The dielectric constant is known to vary by a small amount with temperature and pressure of the liquid ^{3}He. In Supplementary Fig. **12** we show the measured frequency shift of the 5.85 GHz resonator as a function of ^{3}He thickness covering the sample. In one case the cell is completely filled, and in the other case it is covered with 10 monolayers of ^{3}He (corresponding to $\sim 4\,$nm), as estimated from the amount of ^{3}He injected and the sinter surface area. We also show the expected frequency shift of the resonator assuming $\varepsilon_r = 1.0426$, calculated using COMSOL electrostatic simulations and assuming a substrate (sapphire) dielectric constant $\varepsilon_r = 10.4$. Frequency shift data is in agreement with simulation within one part in 1000.

We estimate the ^{3}He loss tangent from the single photon internal loss of our resonators. We fit the extracted TLS-limited internal Q to the empirical formula

$$Q_{\text{int}}^{-1} = \frac{F \tan\delta}{(1 + \langle N \rangle / n_c)^{\alpha}}, \tag{8}$$

where $\langle N \rangle$ is the average photon number in the resonator, and n_c is a critical photon number that depends on the TLS coupling strength to the resonator and their relaxation times. The standard tunneling model [11] predicts an exponent $\alpha = 0.5$ but in high Q superconducting resonators a value of $\alpha < 0.5$ is often found, a signature of TLS-TLS interactions [12]. We summarise relevant extracted numbers in Supplementary Table **1**.

There is no noticeable effect of ^{3}He on the single photon Q_i, instead the variations seen can be attributed to temporal drift due to TLS. Based on this we conclude that the loss introduced by ^{3}He is much smaller than the fluctuations, and we take the mean of all observations and evaluate the upper limit for $F \tan\delta \ll (1/\langle Q_i^{\text{full}} \rangle - 1/\langle Q_i^{\text{empty}} \rangle)^{-1} = 1.5 \times 10^{-6}$. We note that the error intervals reported are errors from the fits and do

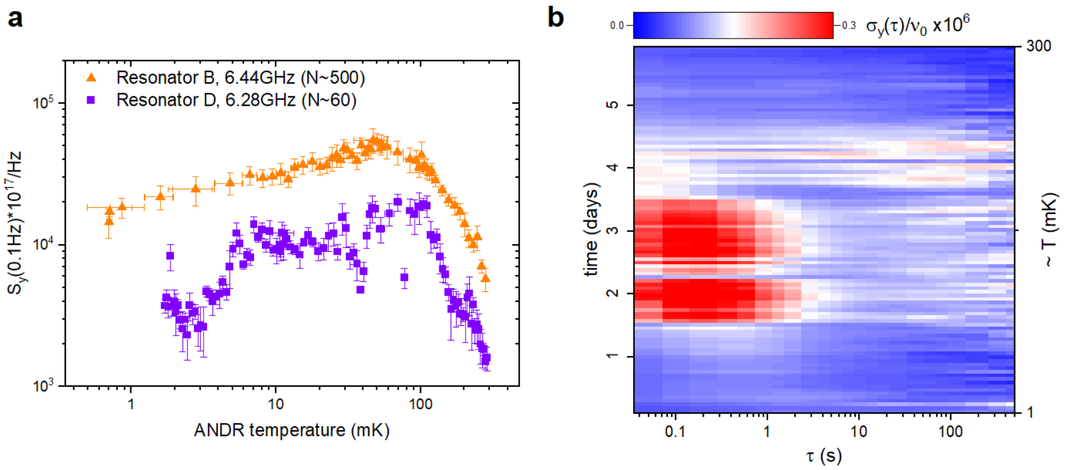

Supplementary Fig. 9. Noise in resonator D. a) the temperature dependence of the noise in the presence of ^{3}He. For reference we also compare with the data for resonator B also presented in Figure 3 of the main manuscript. Error bars are defined as described in Supplementary Note 3. b) The calculated $\sigma_y(\tau)$ for the whole temperature ramp in a). Color scale is magnitude of the AVAR $\sigma_y(\tau)$ with blue corresponding to low values and red to higher values.

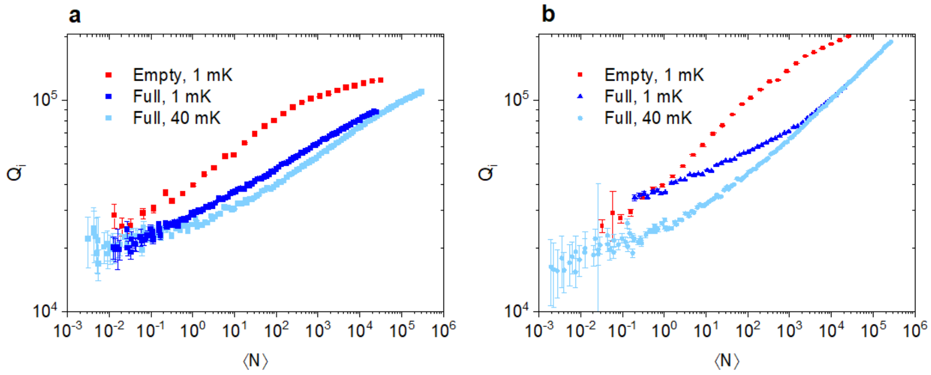

Supplementary Fig. 10. Measured internal quality factor of a) resonator A (5.85 GHz) and b) resonator B (6.45 GHz) versus the average number of photons in the resonator. Resonator B was here subject to significant temporal fluctuations in the single photon Q_i due to TLS. Error bars for all panels are 95% confidence bounds from fits to experimental data.

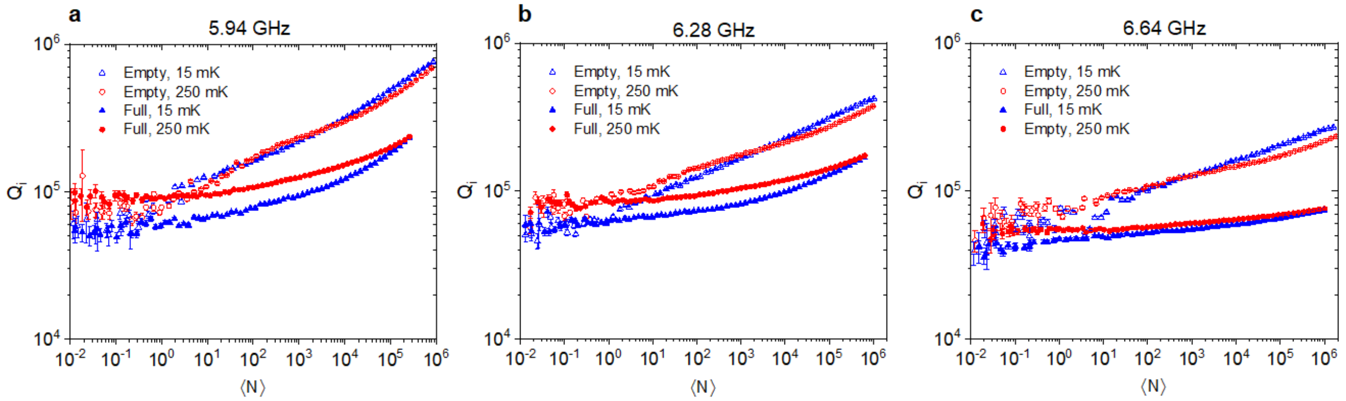

Supplementary Fig. 11. Internal Quality factor versus average photon number for the three resonators C, D, and E, with and without ^{3}He for two temperatures. Error bars for all panels are 95% confidence bounds from fits to experimental data.

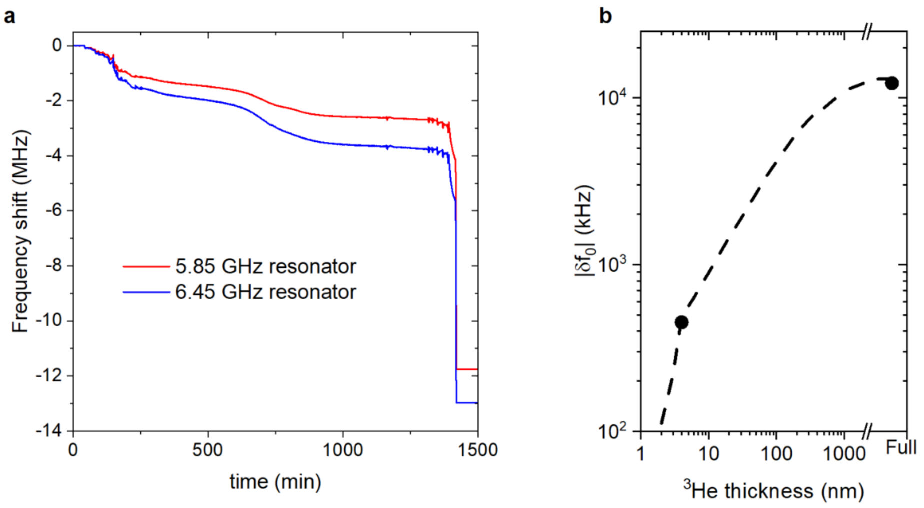

Supplementary Fig. 12. Filling ^{3}He. a) The change in center frequency of two resonators as the cell is filled with ^{3}He, by condensing from the room temperature gas handling system with the cell held at around 300 mK. The sharp drop in frequency provided a clear signature of the cell filling with sufficient liquid to cover the entire mode volume of the resonators. b) Expected frequency shift versus ^{3}He film thickness, confirming this observation. Markers are measured resonator frequency shift of the 5.85 GHz resonator in the presence of different amounts of ^{3}He and the dashed line is the expected frequency shift obtained from COMSOL electrostatic simulations assuming a dielectric constant of ^{3}He of 1.0426.

	Δt (days)	f_0 (MHz)	$Q_{i,n=1}/10^4$ $(F \tan\delta)^{-1}$	$Q_c/10^4$
Resonator A, empty 330mK	0	5839	3.7 ± 0.2	6.8 ± 0.1
Resonator A, empty 5mK	0	5839	3.2 ± 0.2	6.8 ± 0.1
Resonator A, empty 1mK	1	5839	3.4 ± 0.2	6.6 ± 0.2
Resonator A, full 1mK	13	5839 - 12.2	2.5 ± 0.2	7.1 ± 0.1
Resonator A, full 1mK	44	5839 - 12.2	2.0 ± 0.1	7.1 ± 0.1
Resonator A, full 40mK	45	5839 - 12.2	2.2 ± 0.1	7.3 ± 0.3
Resonator A, full 40mK	47	5839 - 12.2	2.3 ± 0.1	7.6 ± 0.5
Resonator A, full 200mK	59	5839 - 12.2	3.0 ± 0.1	7.2 ± 0.3
Resonator A, empty 15mK	66	5839	2.8 ± 0.1	7.0 ± 0.9
Resonator A, empty 15mK	67	5839	2.8 ± 0.1	6.9 ± 0.6
Resonator B, empty 330mK	0	6449	3.9 ± 0.2	4.1 ± 0.1
Resonator B, empty 5mK	0	6449	3.0 ± 0.3	4.1 ± 0.1
Resonator B, empty 1mK	1	6449	2.9 ± 0.3	4.1 ± 0.1
Resonator B, full 1mK	13	6449 - 13.5	3.7 ± 0.2	4.1 ± 0.1
Resonator B, full 1mK	44	6449 - 13.5	2.3 ± 0.3	4.1 ± 0.1
Resonator B, full 40mK	45	6449 - 13.5	2.1 ± 0.1	4.2 ± 0.2
Resonator B, full 40mK	47	6449 - 13.5	2.3 ± 0.1	4.5 ± 0.4
Resonator B, full 200mK	59	6449 - 13.5	3.0 ± 0.1	4.2 ± 0.1
Resonator B, empty 15mK	66	6449	2.3 ± 0.1	4.3 ± 0.8
Resonator B, empty 15mK	67	6449	1.9 ± 0.1	4.3 ± 0.8
Resonator C, empty 15mK	0	5947.6	6.6 ± 0.8	27 ± 0.4
Resonator C, full 15mK	8	5947.6-12.8	6.0 ± 0.2	23 ± 0.4
Resonator C, full 15mK	8	5947.6-12.8	7.4 ± 0.2	23 ± 0.4
Resonator D, empty 15mK	0	6285.6	5.4 ± 0.3	10.4 ± 0.2
Resonator D, full 15mK	8	6285.6-13.5	6.4 ± 0.2	11.4 ± 0.2
Resonator D, full 15mK	8	6285.6-13.5	6.3 ± 0.1	11.2 ± 0.2
Resonator E, empty 15mK	0	6658.1	5.4 ± 0.4	15.7 ± 0.9
Resonator E, full 15mK	8	6658.1-14.1	4.1 ± 0.2	8.0 ± 0.2
Resonator E, full 15mK	8	6658.1-14.1	4.5 ± 0.1	7.5 ± 0.2

Supplementary Table 1. Extracted resonator parameters with and without ^{3}He. Ranges given are the 95% confidence bounds from fits to Supplementary Eq. (8). Intrinsic quality factors are reported for single photon population and coupling quality factors are average across low power measurements.

not capture the temporal variations in the parameters.

Next we estimate the filling factor which will allow us to put a bound on the loss tangent. Using COMSOL for electrostatic simulations of the electric field magnitude in the relevant dielectric volumes we obtain

$$F = \frac{\int_{V_{^3\mathrm{He}}} \varepsilon_r |E^2| dV}{\int_{V_{^3\mathrm{He}}} \varepsilon_r |E^2| dV + \int_{V_s} \varepsilon_s |E^2| dV} = 0.10. \quad (9)$$

Here s denotes the substrate and we use $\varepsilon_s = 10.4$ for the sapphire substrate and $\varepsilon_r = 1.0426$ for ^{3}He. We thus arrive at $\tan\delta \ll 1.5 \times 10^{-5}$.

For a qubit with relaxation limited by dielectric loss we have $T_1 = \nu_{\mathrm{qubit}} F_{\mathrm{qubit}} \tan\delta$. If ^{3}He fills the whole volume above the qubit and using a substrate with similar dielectric constant, then $F_{\mathrm{qubit}} \approx F$, and hence we find a lower bound imposed by the dielectric loss in ^{3}He on qubit coherence of $T_1 > 110$ μs for $\nu_{\mathrm{qubit}} = 6$ GHz.

D. Cooling in the presence of a thin ^{3}He film

A frequency shift of 450 kHz was observed for resonator A as a small amount of ^{3}He was first condensed in the sample cell at 300 mK. From the amount of ^{3}He injected and the sinter surface area we estimate a thin film of approximately 10 atomic layers ($\sim$ 4 nm) covering the surface of the sample. The 450 kHz shift is 3.7% of the total frequency shift seen when fully immersed. This is in good agreement with electrostatic simulations of the resonator frequency shift assuming a dielectric constant of ^{3}He of 1.0426, as we show in Supplementary Fig. **12**.

Supplementary Fig. **13** compares noise measured with a thin ^{3}He film coverage against the noise in the completely filled cell. For the thin ^{3}He film the magnitude of the noise is constant below $T \sim 100$ mK, the same as for the empty cell. The data for the filled cell is in good agreement for the trend observed also for resonator B (see main text).

It thus appears that just a small amount of ^{3}He does not cool down the TLS bath sufficiently to observe any clear change in the noise compared to vacuum. However, the drastic change in saturation power (measured from the power dependence of the resonator) is still observed even with a small amount of ^{3}He present, shown in Supplementary Fig. **14**. This is consistent with the picture of ^{3}He strongly interacting with the TLS, increasing their energy relaxation. To the contrary, with just a thin film of ^{3}He the cooling of the ^{3}He itself is expected to be very poor, and there may not even be a continuous thermal link to the sinter heat exchangers and the ANDRP.

We also investigated the cooling of surface spins with the same small amount of ^{3}He present. Supplementary Fig. **15** shows the temperature dependence of the second hydrogen peak which is expected to have a population and peak intensity vanishing as temperature goes to zero. Here we find that even a small amount of ^{3}He is enough to cool the surface spins.

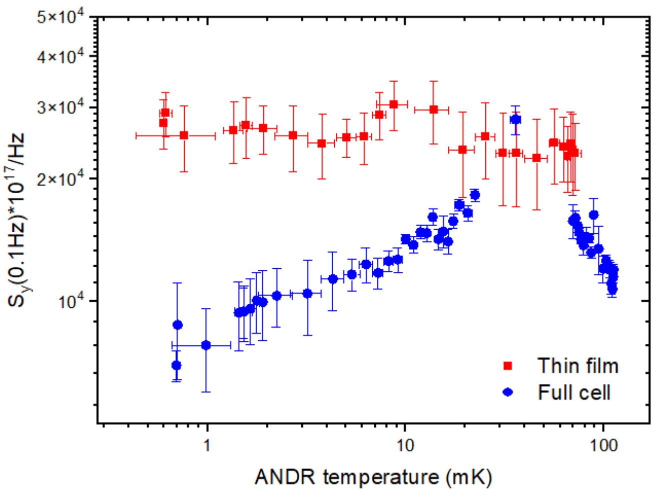

Supplementary Fig. 13. Noise for resonator A (5.85 GHz) compared with cell filled completely with ^{3}He and with just a thin layer on the surface. In both cases $\langle N \rangle \sim 400$. Gaps in the data for the full cell is due to a hardware fault during this time. Error bars are as defined in Supplementary Note 3.

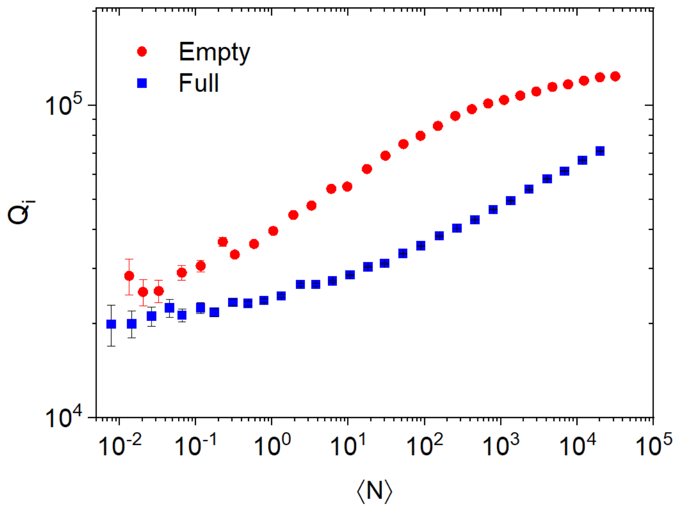

Supplementary Fig. 14. Internal Quality factor versus average photon number for resonator A (5.85 GHz) in the empty (vacuum) cell and with just a thin layer of ^{3}He on the surface. Measured with a ANDRP temperature of 1 mK. Error bars are 95% confidence bounds from fits to experimental data.

E. Pressure dependence

Experiments at elevated pressures of ^{3}He were conducted at 10 mK. We track the resonance frequency as we apply pressure, and plot the change in frequency in Supplementary Fig. **16**a. From the measured dielectric constant and the known change in ^{3}He density and speed of sound [6] we can calculate the expected frequency shift using the Clausius-Mossotti relation. This is found to be

From dataset	-1-2μ for $T > T_x$
Resonator A, Full	-1.50 ± 0.9
Resonator B, Full	-1.76 ± 0.18
Resonator B, Empty	-1.47 ± 0.12
Resonator B, Empty	-1.42 ± 0.16
From dataset	T^{β} for $T < T_x$
Resonator A, Full	0.25 ± 0.04
Resonator B, Full	0.24 ± 0.005

Supplementary Table 2. Summary of extracted values for the parameter μ from different resonators and parts of data. Low T refers to fits to the noise at temperatures $T < 20$ mK in the saturated TLS regime, and high T to the region $90 < T < 250$ mK where the scaling $T^{-1-2\mu}$ is expected to hold, this range is avoiding the cross-over region and not exceeding $hf > k_b T$.

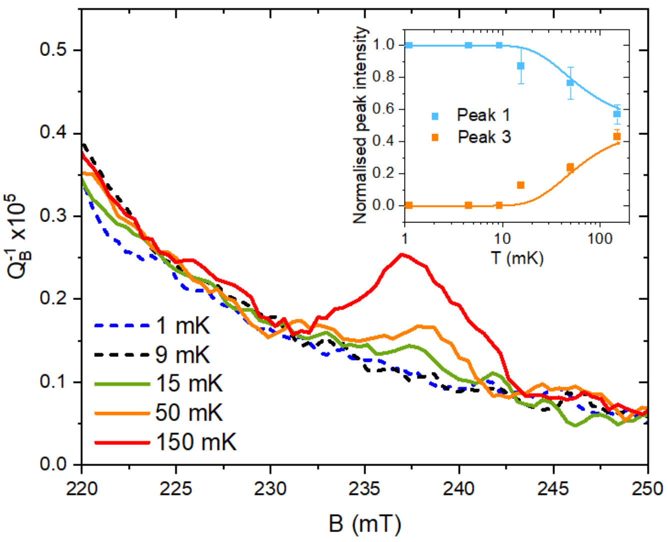

Supplementary Fig. 15. ESR peak intensity for the high-field atomic hydrogen peak versus nuclear stage temperature in the presence of a thin ^{3}He film on the sample surface. $\langle N \rangle \sim 10^3$. The inset shows the fitted normalised peak intensity and the expected intensity due to thermal population of the ESR levels split by 1.42 GHz. Error bars are propagated errors from fitting the peak intensities. At 1, 5 and 9 mK no trace of a peak is observed above the noise and datapoints are set to zero.

in excellent agreement with experimental data, confirming that the applied pressure is what we expect. In Supplementary Fig. **16**b we then show the internal quality factor of the resonator versus average photon number, and compare it to the case of zero pressure. We find that increasing the pressure to 5 bar has a very small effect on the saturation power, increasing it about 20%, a precise number is hard to estimate due to TLS parameter drift. Measurements at still higher pressure were not possible due to limitations of our immersion cell design.

F. Additional data on noise at sub-mK temperatures

Supplementary Fig. **17** shows a separate measurement where the sample was allowed to very slowly warm up from the lowest possible temperature of $\sim$ 400 μK up to $\sim$ 2 mK over the course of 8 days. Supplementary Fig. **17**a shows the extracted magnitude of the 1/f noise and panel b shows the whole AVAR dataset. Even at these low temperatures TLS temporal fluctuations are evident as "bumps" appearing and disappearing on various timescales.

While we cannot completely rule out TLS temporal drift, this additional data in Supplementary Fig. **17** shows a clear increase of the noise starting at $T \sim 0.8$ mK. Below this temperature the noise remains constant with temperature, likely due to inefficient thermalisation. This also means that the low temperature increase in noise in the $N = 20$ data presented in Figure 3a in the main manuscript is due to TLS temporal drift. The $\sim$ 1 mK saturation temperature is close to the superfluid transition of ^{3}He occurring at $T_c = 0.9$ mK at saturated vapour pressure. Hence the observed saturation at low T may suggest that the cooling of the low energy fluctuators becomes less efficient once ^{3}He enters the superfluid state. However, we expect this transition to be smooth and the effect to be small near T_c, as the BCS gap in the ^{3}He spectrum that opens up scales as $\Delta_{^3\mathrm{He}} = 3.06 k_b T_c (1 - T/T_c)^{1/2}$.

5. THEORETICAL MODELS

A. Temperature dependence of the noise

A detailed derivation of the theory for the generalised tunneling model (GTM) of interacting TLS at low temperatures can be found in [13]. Here we give a brief summary of the GTM main result, the temperature dependence of the resonator frequency noise, which is valid in the regime $\Gamma_1 \ll \Gamma_2$ for the TLS bath. The model partitions the TLS in two categories: those that are high energy coherent fluctuators with energies $> k_B T$ and some of these are also (near-)resonant with the res-

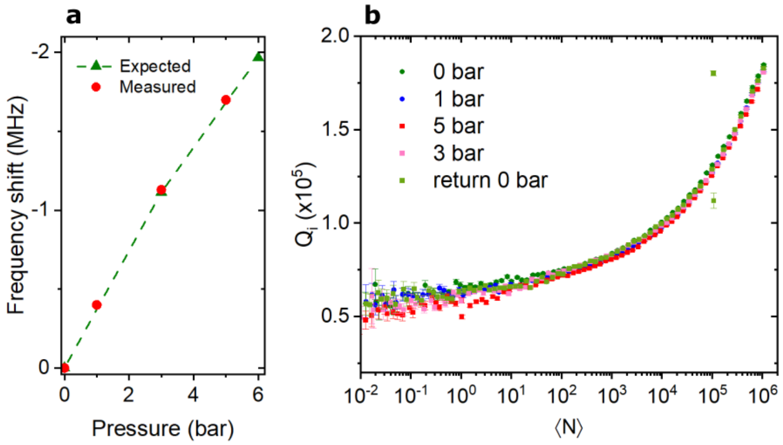

Supplementary Fig. 16. a) Increasing the ^{3}He pressure modifies the dielectric constant according to the Clausius-Mossotti relation. Using tabulated data for ^{3}He density and speed of sound, and a zero pressure dielectric constant $\varepsilon_r = 1.0426$ we calculate the expected frequency shift as a function of applied pressure. The expected frequency shift is in good agreement the measured shift, thus confirming that the ^{3}He pressure in the cell matches that at room temperature where the pressure gauge is located. Data obtained at 10 mK for the 6.26 GHz resonator. b) Internal Quality factor vs average photon number for the same resonator for pressures up to 5 bar. A small change my be present, but we cannot exclude the possibility that this is due to parameter fluctuations. Error bars are 95% confidence bounds from fits to experimental data.

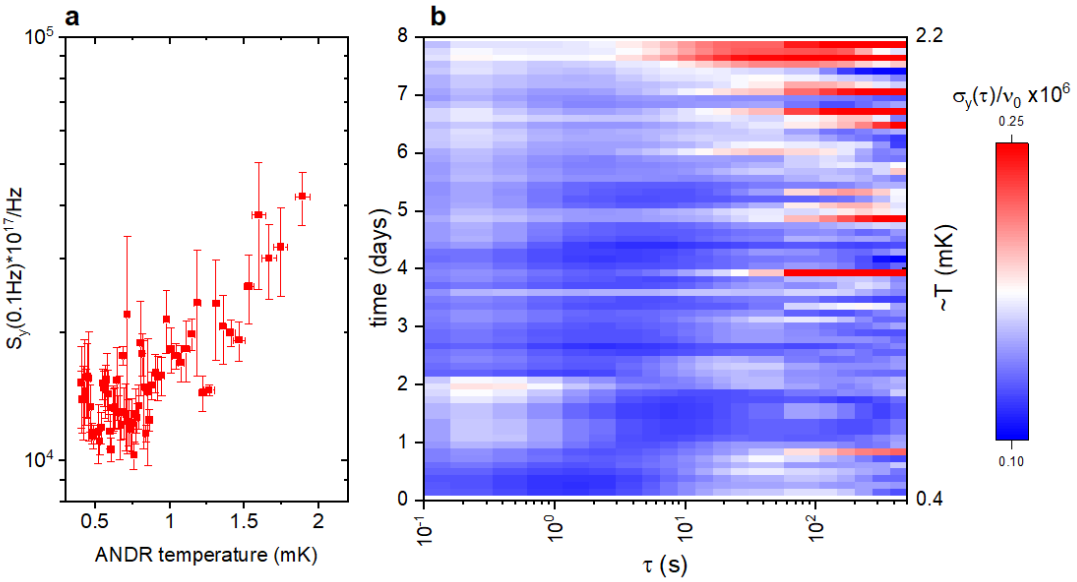

Supplementary Fig. 17. a) A slow warmup experiment exploring the lowest accessible temperatures. $\langle N\rangle \sim 400$. b) The calculated $\sigma_y(\tau)$ across all measured timescales, highlighting the temporal drift of individual TLS at different timescales. Error bars are as defined in Supplementary Note 3.

onator. These couple to the resonator and absorb energy (which is then dissipated to phonons in the bulk via the TLS relaxation rate Γ_1). Thermal fluctuators are those TLS with an energy smaller than temeprature $E \ll k_B T$. The random telegraph switching of these fluctuators contribute to the frequency noise if they strongly perturb a nearby coherent TLS, or they can contribute to the dephasing rate Γ_2 of the TLS if weakly coupled [13].

The low energy thermal fluctuators are assumed to have a poissonian probability distribution of switching rates γ and a uniform energy distribution $\rho^F(E,\gamma) = \rho_0^F/\gamma$. A strongly coupled fluctuator is considered activated, i.e. contributing to the noise and undergoing random telegraph switching, if the temperature exceeds its energy scale. Hence the number of activated fluctuators scales as $\int_0^T \rho_0^F dE = \rho_0^F T$. The average number of activated fluctuators coupled to each resonant TLS is then given by $\mathcal{N}_F(T) = \frac{4\pi}{3}\rho_0^F R_0^3 T$. Here R_0 is the interaction radius of the TLS, i.e. a fluctuator inside this

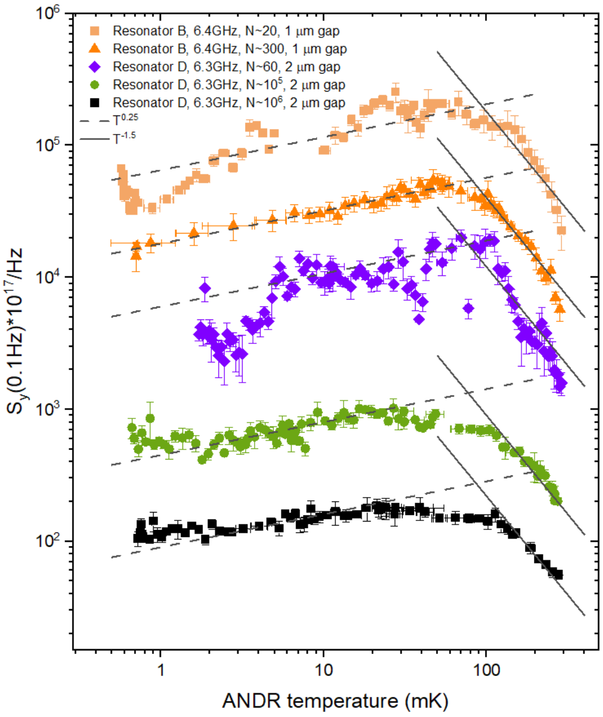

Supplementary Fig. 18. $1/f$ frequency noise as a function of temperature with the cell filled with ^{3}He. Comparison for several driving strengths ranging from $N \sim 60$ to $N \sim 10^6$ and for two different capacitor gap sizes in the resonators that otherwise have nominally the same frequency. The data shows that the crossover temperature T_x remains the same under all these conditions and that the temperature dependence below T_x appears universal. Several of the datasets the same as in other figures presented, here reproduced for convenience. Error bars are as defined in Supplementary Note 3.

radius couples strongly such as to shift the TLS energy more than its linewidth.

We note that in [13] $\mathcal{N}_F$ was estimated to be ~ 1 and this term dropped. Subsequent experiments [14–16] revealed that in fact $\mathcal{N}_F \gg 1$ which means the magnitude of the $1/f$ noise acquires another temperature dependent prefactor, $\mathcal{N}_F$.

The frequency noise spectrum is defined as the autocorrelation of frequency fluctuations in the frequency domain

$$\frac{S_{\delta\nu}}{\nu_0^2} = \lim_{\tau\to\infty}\frac{1}{\tau}\int_0^\tau\int_0^\tau \frac{\langle\delta\nu(t_1)\delta\nu(t_2)\rangle}{\nu_0^2} e^{i\omega(t_1-t_2)dt_1dt_2}, \tag{10}$$

where ν_0 is the resonance frequency. Evaluating this in the limit of low temperature $k_bT \ll h\nu_0$ and including the contribution from multiple fluctuators coupled to each TLS gives

$$\frac{S_{\delta\nu}}{\nu_0^2}(\omega) \sim \frac{8}{15}\langle d_0^4\rangle \frac{P_\gamma}{\omega}\frac{\chi}{U_0\Gamma_2}\mathcal{N}_F(T)F(\mathcal{E}), \tag{11}$$

with

$$F(\mathcal{E}) = \left(\int_{V_h} dV \frac{|\mathcal{E}|^4}{\sqrt{1+|\mathcal{E}/\mathcal{E}_c|^2}}\right) / \left(\int_V \epsilon|\mathcal{E}|^2 dV\right)^2. \tag{12}$$

We further have that the TLS linewidth is given by

$$\Gamma_2 = c_0\chi \ln\left(\frac{\Gamma_1^{\max}}{\Gamma_1^{\min}}\right)\frac{T^{1+\mu}}{\nu_0^\mu}, \tag{13}$$

where $\Gamma_1^{\max}$ and $\Gamma_1^{\min}$ is the maximum and minimum phonon relaxation rates of the distribution of TLS in the bath [13]. This temperature dependence of the linewidth arises from the TLS interactions. Furthermore,

$$\chi = P_0U_0\left(\frac{\nu_0}{E_{\max}}\right)^\mu \approx \tan\delta_i, \tag{14}$$

which is independent of temperature and

$$\mathcal{E}_c = \frac{\sqrt{\Gamma_1\Gamma_2}}{2\langle d_0|\sin\theta|\rangle} \tag{15}$$

is the critical electric field for saturation which inherits a temperature dependence only from Γ_2 such that $\mathcal{E}_c(T) \sim T^{(1+\mu)/2}$. d_0 is the TLS dipole moment and θ their relative angle to the local microwave electric field from the device. The critical photon number is proportional to the critical field squared: $N_c \propto \mathcal{E}_c^2(T) \propto \Gamma_1\Gamma_2$, which thus acquires a temperature dependence $T^{1+\mu}$.

Taken together we can write the temperature dependent contributions to the resonator $1/f$ frequency noise as

$$\frac{S_{\delta\nu}}{\nu_0^2}(\omega, T) \sim \frac{1}{\omega}\frac{T}{\Gamma_2}R_0^3F(\mathcal{E}). \tag{16}$$

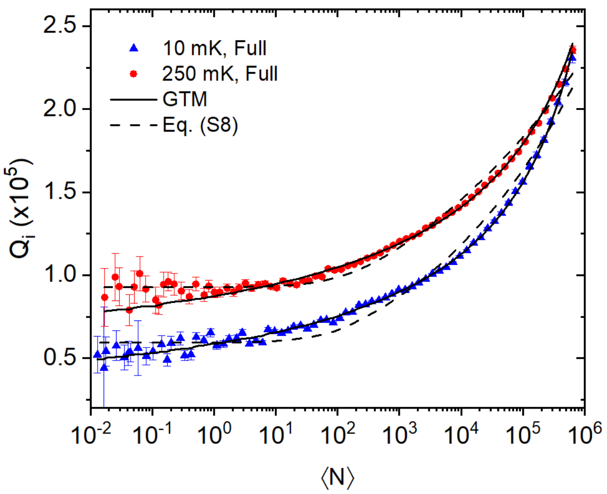

Supplementary Fig. 19. Comparison of fits to Supplementary Eq. (8)) with $\alpha = 0.11$, and GTM (Supplementary Eq. (19)) for two temperatures with the cell filled with ^{3}He. Error bars are 95% confidence bounds from fits to experimental data.

For weak driving fields, $\mathcal{E}/\mathcal{E}_c \ll 1$ $F(\mathcal{E})$, becomes a temperature independent constant prefactor and TLS have a spectral width Γ_2, such that $R_0^3 = U_0/\Gamma_2$. In the strong field regime the TLS are power broadened such that instead $R_0^3 = U_0/\Omega_R$, where $\Omega_R = 2\sin\theta d_0 \cdot \mathcal{E}$ is the Rabi frequency and $U_0 = d_0^2/\varepsilon$ is the dipole-dipole interaction strength. In the strong field regime we have also that $F(\mathcal{E}) \propto \mathcal{E}_c$. Taken together

$$\frac{S_{\delta\nu}}{\nu_0^2}(\omega, T) \sim \begin{cases} \frac{1}{\omega}\frac{T}{\Gamma_2^2} \propto T^{-1-2\mu} & \text{for} \quad \mathcal{E} \ll \mathcal{E}_c \\ \frac{1}{\omega}\frac{T\mathcal{E}_c}{\Gamma_2} \propto T^{(1-\mu)/2} & \text{for} \quad \mathcal{E} \gg \mathcal{E}_c. \end{cases} \tag{17}$$

Hence, for a constant driving power, starting at high temperatures and weak fields, we expect a crossover into the strong fields regime as we cool down the TLS bath and the TLS coherence increases, provided that $\Gamma_1 \ll \Gamma_2$ at high temperature. With $\mu = 0.25$ as we extract at high temperatures this would yield a low temperature power saturation scaling as $T^{0.375}$, somewhat different from what we observe. It can also be understood that the crossover temperature T_x acquires a power dependence $T_x \propto \langle N\rangle^{1/(2+2\mu)}$, such that if we increase the driving power, the crossover is expected to also move to higher temperatures. Likewise if the electric field strength in the device is diluted (by increasing the gap between metal electrodes in the resonator) T_x should scale accordingly with electric field strength. In Supplementary Fig. **18** we compare several measurements varying the driving power by five orders of magnitude and and the capacitor gap by a factor 2, none of which has any effect on the crossover temperature, implying that TLS saturation cannot fully explain our results.

Contrary to Supplementary Eq. 17, if we instead are in the limit where relaxation dominates the TLS linewidth (for $T < T_x$) the GTM predicts

$$\frac{S_{\delta\nu}}{\nu_0^2}(\omega, T) = \frac{1}{\omega}\frac{T}{\Gamma_1}R_0^3 F(\mathcal{E}) \propto T, \tag{18}$$

for all $\mathcal{E}$, assuming that Γ_1 is independent of temperature. I.e. in this regime the model does not match the observed $T^{0.25}$ trend.

Both in the relaxation limited regime and in the original GTM regime of $\Gamma_1 \ll \Gamma_2$ we expect a power dependence of the noise according to $S_y \propto (1 + \langle N\rangle/N_c)^{-1/2}$, as we also observe in Figure 3b.

Finally we note that our conclusion that ^{3}He only affects Γ_1 is consistent with the expected effect that ^{3}He has on the TLS elastic phonon interaction energy $U_0^{ph} \approx M^2/\rho v^2$. Using the values for M, ρ, and v for sapphire and ^{3}He quoted in the main manuscript we estimate $U_0^{He}/U_0^{sap} \approx 0.2$. This means that dipolar interactions still dominate over elastic interactions in the presence of ^{3}He, in agreement with the fact that we observe no change in the interaction parameter μ nor the single photon $Q_i \propto \chi^{-1} \propto U_0^{-1}$.

B. Power dependence of the quality factor

Significant TLS interactions yield a logarithmic dependence of the quality factor on photon number [12, 14]

$$\frac{1}{Q_\text{i}} = P_\gamma F \tan\delta \ln\left(c\sqrt{\frac{N_c}{\langle N\rangle}}\right). \tag{19}$$

Here $F\tan\delta$ is the single photon loss tangent, P_γ is a constant of order one arising from the distribution of fluctuator switching rates, and c is a constant quantified in [13].

Supplementary Fig. **19** compares typical $Q_i(\langle N\rangle)$ data with fits to the empirical formula Supplementary Eq. (8), and to the GTM expectation, Supplementary Eq. (19). There is exceptionally good agreement to the logarithmic GTM result, except at the very lowest photon numbers where noisy data obscures detailed comparison. Past comparison between these two models has often been challenging at mK temperatures, neither capturing exact dependencies precisely, which is still true for the vacuum measurements performed here. A reason for this is likely due to non-equilibrium effects and the interplay between overheating of the TLS bath and power saturation. In the presence of ^{3}He we now achieve very good agreement, likely because the system is much closer to thermal equilibrium throughout the whole power range. We also note that for the evaluation of $F\tan\delta$ and the single photon loss values in Supplementary Table **1** we still use Supplementary Eq. (8) for fits to remove the ambiguity from estimating the parameter P_γ.

[1] J. Nyéki, M. Lucas, P. Knappová, L. V. Levitin, A. Casey, J. Saunders, H. van der Vliet, and A. J. Matthews, Phys. Rev. Applied **18**, L041002 (2022).

[2] C. Lusher, J. Li, V. Maidanov, M. Digby, H. Dyball, A. Casey, J. Nyéki, V. Dmitriev, B. Cowan, and J. Saunders, Measurement Science and Technology **12**, 1 (2001).

[3] A. Shibahara, O. Hahtela, J. Engert, H. van der Vliet, L. Levitin, A. Casey, C. Lusher, J. Saunders, D. Drung, and T. Schurig, Philosophical Transactions of the Royal Society A: Mathematical, Physical and Engineering Sciences **374**, 20150054 (2016).

[4] S. Krinner, S. Storz, P. Kurpiers, P. Magnard, J. Heinsoo, R. Keller, J. Luetolf, C. Eichler, and A. Wallraff, EPJ Quantum Technology **6**, 2 (2019).

[5] F. Pobell, *Matter and methods at low temperatures*, Vol. 2 (Springer, 2007).

[6] R. Dobbs, *Helium Three* (OUP Oxford, 2001).

[7] S. Mahashabde, E. Otto, D. Montemurro, S. E. de Graaf,

S. E. Kubatkin, and A. Danilov, Phys. Rev. Applied **14**, 044040 (2020).
[8] S. E. de Graaf, D. Davidovikj, A. Adamyan, S. E. Kubatkin, and A. V. Danilov, Appl. Phys. Lett. **104**, 052601 (2014).
[9] T. Lindström, J. Burnett, M. Oxborrow, and A. Y. Tzalenchuk, Review of Scientific Instruments **82**, 104706 (2011).
[10] E. Rubiola, *Phase noise and frequency stability in oscillators* (Cambridge University Press, 2009).
[11] W. A. Phillips, Rep. Prog. Phys. **50**, 1657 (1987).
[12] L. Faoro and L. B. Ioffe, Phys. Rev. Lett. **109**, 157005 (2012).
[13] L. Faoro and L. B. Ioffe, Phys. Rev. B **91**, 014201 (2015).
[14] J. Burnett, L. Faoro, and T. Lindstrom, Superconductor Science and Technology **29**, 044008 (2016).
[15] S. E. de Graaf, L. Faoro, J. J. Burnett, A. A. Adamyan, S. E. Kubatkin, A. Y. Tzalenchuk, T. Lindström, and A. V. Danilov, Nature Communications **9**, 1143 (2018).
[16] S. E. de Graaf, S. Mahashabde, S. E. Kubatkin, A. Y. Tzalenchuk, and A. V. Danilov, Phys. Rev. B **103**, 174103 (2021).
